# Migratory strategies across an ecological barrier: is the answer blowing in the wind?

**DOI:** 10.1186/s40462-024-00509-2

**Published:** 2024-10-14

**Authors:** Rosalyn E. Bathrick, James A. Johnson, Daniel R. Ruthrauff, Rebekah Snyder, Maria Stager, Nathan R. Senner

**Affiliations:** 1 Organismic and Evolutionary Biology Program, University of Massachusetts Amherst, Amherst, MA USA; 2 U.S. Fish & Wildlife Service, Migratory Bird Management, Anchorage, AK USA; 3 U.S. Geological Survey, Alaska Science Center, Anchorage, AK USA; 4 Department of Environmental Conservation, University of Massachusetts Amherst, Amherst, MA USA; 5 Department of Biology, University of Massachusetts, Amherst, Amherst, MA USA

**Keywords:** Ecological barrier, Migratory divide, Wind, Southbound migration

## Abstract

**Background:**

Ecological barriers can shape the movement strategies of migratory animals that navigate around or across them, creating migratory divides. Wind plays a large role in facilitating aerial migrations and can temporally or spatially change the challenge posed by an ecological barrier, with beneficial winds potentially converting a barrier into a corridor. Here, we explore the role wind plays in shaping initial southbound migration strategy among individuals breeding at two sites along an ecological barrier.

**Methods:**

Using GPS satellite transmitters, we tracked the southbound migrations of Short-billed Dowitchers *(Limnodromus griseus caurinus)* from two breeding sites in Alaska to nonbreeding sites in coastal Mexico. The breeding sites were positioned in distinct regions along an ecological barrier – the Gulf of Alaska. We investigated potential differences in migratory timing, wind availability, and tailwind support *en* route across the Gulf of Alaska between individuals breeding at the two sites.

**Results:**

Route choice and arrival timing to wintering sites differed markedly between the two breeding sites: individuals departing from the more westerly site left at the same time as those from further east but crossed the Gulf of Alaska farther west and arrived along the Pacific coast of Mexico an average of 19 days earlier than their counterparts. Dowitchers from both sites departed with slight tailwinds, but once aloft over the Gulf of Alaska, birds from the more westerly site had up to twelve times more tailwind assistance than birds from the more easterly one.

**Conclusions:**

The distinct migration strategies and degree of wind assistance experienced by birds at these two breeding sites demonstrates how differences in wind availability along migratory routes can form the basis for intraspecific variation in migration strategies with potential carryover effects. Future changes in wind regimes may therefore interact with changes in habitat availability to influence migration patterns and migratory bird conservation.

## Background

Migratory animals confront a multitude of environmental conditions and landscapes throughout their annual cycles. These conditions change over both short- and long-term timeframes, shaping inter- and intraspecific route choices, sites used in transit, and movement timing. The existence of distinct migratory strategies, whether over the course of an individual’s life or among individuals within a population, is often caused by enduring ecological barriers where organisms are physiologically limited (e.g., unable to rest or refuel). For example, the Gulf of Mexico [1], Sahara Desert [2], and Tibetan Plateau [3] all represent large-scale obstacles that impede the direct passage of avian migrants and have created migratory divides [4] – disjunctions that lead geographically adjacent groups of a species to use different routes to migrate to their subsequent destinations [3, 5, 6]. While these barriers may appear formidable for most avian migrants, seasonal and variable abiotic conditions like weather can play a dynamic role in facilitating passage across them [7]. Animals capable of capitalizing on certain conditions may be able to use an apparent barrier as a beneficial corridor [8], for instance, giving them an advantage over those that are forced to avoid the barrier altogether. As abiotic conditions increase in variability globally, identifying the role that they currently play in driving differences in migratory strategies around and across barriers can illuminate the tradeoffs faced by migratory animals.

Wind, in particular, influences aerial movements and migration strategies in diverse ways [9]. In the case of long-distance migrations, wind frequently plays a direct and crucial role [10]. The availability of tailwinds can influence migratory departure dates in songbirds [11], facilitate passage over open oceans for shorebirds [12, 13], and support raptor movements between island stopovers [14]. However, access to beneficial winds might differ across a migratory species’ range, shaped by both proximity to specific geographic features and less predictable intra- and inter-annual variation in conditions [15]. Given the impact wind can have on both migratory phenology and strategy, this differential access can create a gradient of opportunities, dividing populations by their ability to take advantage of winds within or between years. Over time, these distinct localized conditions may drive the use of divergent migratory strategies, resulting in temporal or spatial migratory divides among populations.

One consequence of migratory divides is spatiotemporal differences in habitat use between individuals or populations. The consequences of temporal divides can be varied and indirect, but might result in: mismatched arrival time at breeding grounds, which can lead to assortative mating [16]; phenological mismatches with prey at stopover sites, which can shape body condition and subsequent migratory decision making [17]; or, territorial disputes on the non-breeding grounds [18], which can influence overwinter survival and migratory readiness [19]. For example, Icelandic Black-tailed Godwits (*Limosa limosa islandica*) that breed in recently colonized, lower quality nesting grounds arrive to coastal wintering sites later than their counterparts breeding in higher quality habitats and are pushed onto sites with reduced prey availability [20]. Alternatively, Eurasian Hoopoes (*Upupa epops*) experiencing suboptimal conditions during one portion of their annual cycle can, to an extent, adjust the duration or timing of subsequent periods and minimize potential reversible state effects [21]. The direct consequences of spatial divides, on the other hand, are well documented: some routes can increase exposure to pesticides [22], hunting [23], or limited stopover habitats [24]. The degree to which the viability of a species is impacted by these differences may depend on the threats to which separate populations are exposed and the flexibility of individuals to respond to the challenges those threats impose.

The Pacific Basin is a dynamic migratory “theater” [25] that hosts the longest distance avian migrations on record [26]. The Gulf of Alaska is an ecological barrier at the northern margin of the Pacific, surrounded by mountains that create a backstop to highspeed storms moving northward along the Alaskan coast. Because of the confluence of currents and pressure systems, wind in the Gulf of Alaska exhibits weak cyclonic conditions in the summer with substantial regional differences in speed and direction [27], more so than the northerly (southward direction) wind that dominates the California Current farther south [28]. The degree to which near-shore wind is reflective of conditions in the central Gulf of Alaska varies along the coastline, with more northeasterly locations (near the Cook Inlet) being dominated by slightly onshore winds and more westerly locations (along the Alaska Peninsula) by more offshore winds [27]. As a result, the wind conditions on both the coastline of the Gulf of Alaska and at higher altitudes within it are spatially and temporally heterogenous. Accordingly, avian migrants navigating the Gulf of Alaska encounter different levels of wind support and risk, depending on the timing and location of their route, as well as their ability to rest upon open water [29].

**Fig. 1:**
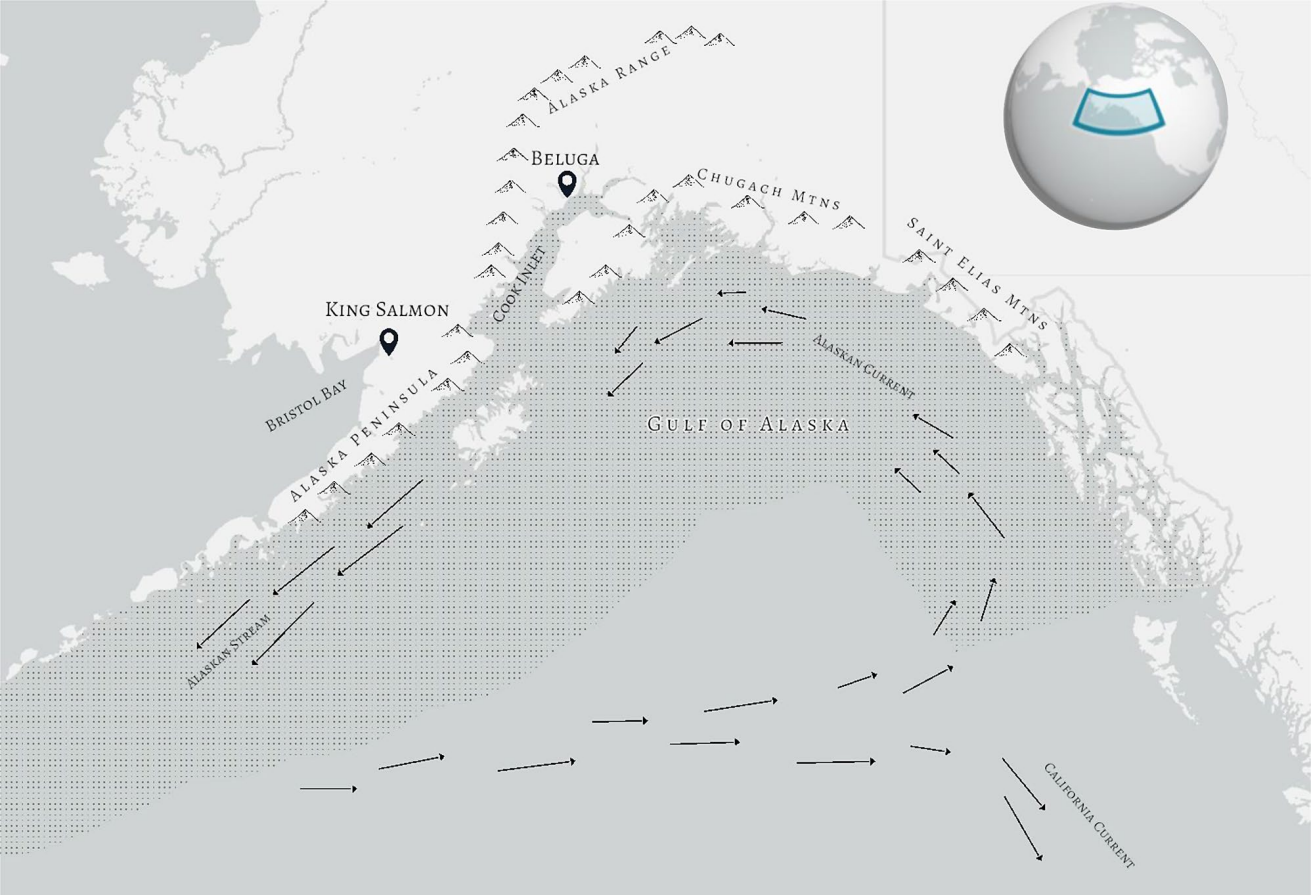
The southern Alaska coast and the Gulf of Alaska, shaded to show boundary. Study sites, King Salmon and Beluga, are separated by the Alaska Range and each positioned near productive tidal mudflats in Bristol Bay and Cook Inlet, respectively. Mountain ranges surround the “graveyard for Pacific Storms”: the Gulf of Alaska, which hosts a confluence of oceanic currents, indicated by arrows

To evaluate the role that dynamic, abiotic conditions might play in shaping avian migrations, we tracked the southbound migration of Alaska-breeding Short-billed Dowitchers (*Limnodromus griseus caurinus*) departing from two breeding sites. Short-billed Dowitchers (hereafter, ‘dowitchers’) are shorebirds that breed along much of the Gulf of Alaska coast. Our study sites were positioned at the western end of their breeding range at the base of the Alaska Peninsula and more centrally in the upper Cook Inlet (Fig. 1). After the breeding season, individuals from both sites migrate south within the Pacific Americas Flyway to the coastlines of Baja California and mainland Mexico [30]. Our objective was to investigate the potential role of wind in influencing the migration strategies of birds at these two sites. We hypothesized that between the two breeding sites there exist differences in: (1) available tailwinds at departure from the breeding grounds, (2) available wind support in flight, with the greatest differences over the Gulf of Alaska, and therefore, (3) southbound migratory strategy. Given these hypotheses, we expected to find that, while the dowitchers breeding on the Alaska Peninsula were afforded less access to potential nearby coastal stopover sites than those in the Cook Inlet region, they were better positioned to capitalize on beneficial winds, especially in the Gulf of Alaska. This would allow them to migrate more directly, shorten their migratory duration, and exhibit both a temporal and spatial migratory divide with the dowitchers from Cook Inlet. This study offers an example of a small-scale migratory divide structured by abiotic, seasonal conditions shaping the temporal connectivity between geographically adjacent populations of the same species. Further, we contextualize the potential tradeoffs posed by these divergent strategies, elucidating conservation implications in the face of globally changing weather patterns and shedding light on the migration strategy of a relatively understudied long-distance migrant.

## Methods

### Study sites

The Gulf of Alaska is bounded by the coast of Alaska to the north and extends south from below Kodiak Island in the West to the Dixon Entrance in the East [31] (Fig. 1). Dowitchers in this study were captured at two sites in the central and western part of the species’ Alaskan breeding range along the Gulf of Alaska: Beluga (*61.208° N*,* -151.017° S*), which is in the upper Cook Inlet in southcentral Alaska, and King Salmon (*58.715° N*,* -156.712° S*), which is located at the base of the Alaska Peninsula in the Bristol Bay watershed. Beluga is positioned at the northernmost edge of the Gulf of Alaska, 160 km from the Pacific coastline but adjacent to productive mudflats that line the coast of the upper Cook Inlet. King Salmon is 130 km from the Gulf of Alaska coastline, neighboring the mudflats of Kvichak Bay. The two sites are 400 km apart and separated by the southwestern portion of the mountains of the Alaska Range, which may act as a potential barrier to gene flow.

### Transmitter deployment and schedule

During the breeding seasons of 2021 and 2022, we captured 32 dowitchers in Beluga (2021: *n* = 12, 2022: *n* = 10) and King Salmon (2022: *n* = 10) with mist nets during nest incubation (*n* = 25) or brood rearing (*n* = 7). Most captured birds were mated pairs that share incubation duties, resulting in a near even ratio of males to females in our sample (14 females, 15 males, and 3 unknown). All 32 birds were fitted with 4.0-g PinPoint Argos 75 GPS satellite transmitters (Lotek Wireless Inc, Ontario, Canada), which were attached with a leg-loop harness using elastic cord (Stretch Magic, Pepperell Co., Pepperell, MA) and jewelry crimps [32]. Combined transmitter and harness material weight did not exceed 5% of a bird’s body mass. Transmitters were programmed to collect GPS-level location data with minimal error (< 10 m) every 2 − 3 days from 1 Jul – 1 Nov to optimize battery life until the end of southbound migration, at which point the schedule changed to a five-day interval between location estimates. Transmitter types differed between years, with some (*n* = 12) transmitters during 2022 equipped to measure altitude to an accuracy of ± 20 m [33], along with GPS locations.

### Flight tracks and stopover sites

Transmitters were synced with Movebank [34] and location classifications were filtered to include those at the 2D and 3D high-accuracy levels, which are derived from ≥ 3 and ≥ 4 satellite messages, respectively [35]. Completeness of individual tracks varied, with occasional unscheduled gaps between fixes. In order to maximize our sample size and make use of partial tracks, we assessed each track for completeness in four different categories. Tracks were assessed for (a) breeding ground departure date with ≤ 3-day gap between points, (b) arrival date to 40.8°N (Northern California) with a ≤ 3-day gap between points, (c) arrival date to 32.7°N, (Southern California) with a ≤ 3-day gap between points and (d) arrival date to the nonbreeding grounds with a ≤ 3-day gap between points. To determine stopover sites for each individual, we identified all locations away from breeding sites where > 2 consecutive points (i.e., over 4 days) were recorded within 20 km of each other. We then calculated stopover duration as the amount of time between the first and last transmission date at the site. We chose 20 km as it encompassed the total area of coastal wetland complexes that were used by dowitchers. This should be considered a minimum for both number of stopovers and stopover duration, as this method did not capture short stopovers < 4 days or stopovers that might have occurred between unscheduled data transmission gaps.

### Migratory timing

To determine departure dates from the two breeding sites, we evaluated the number of days between the final GPS point taken on the breeding grounds and the distance to the first point thereafter (see “day gap” and “km between” in Table 1). For most tags, the number of days between fixes was two, but for three tags, the number of days between fixes ranged from 3 to 4, which increased the number of potential days a bird could have departed. To narrow this window, we used an estimated flight velocity of 18 m/s, which was the calculated maximum groundspeed maintained over the course of 24 h (e.g., 1625 km/day) that we derived from two dowitchers that were tracked for multiple days in flight. Based on the distance from the breeding site a bird flew during the gap between fixes, this flight speed was used to estimate the duration of its first flight, thereby helping to reduce the number of departure day options. Two birds had 5-day gaps between fixes during the departure window. Because the first location away from the breeding site was aloft over the Gulf of Alaska, 1089 km from the breeding grounds, we deduced that those birds departed < 24 h prior to the transmission date. For birds with multiple potential departure days, we ran all models with all departure day options to check for potential differences in weather conditions between days.

We followed a similar method for determining arrival dates to latitudes associated with stopover sites used by individuals from both sites: Arcata Marsh in Northern California (40.8°N), San Diego Bay in southern California (32.7°N), and the final nonbreeding site (latitude varied). If an individual did not use one of these stopover sites, their arrival date was the first date they were tracked south of the latitude associated with each of these sites. Stopover sites farther north than northern California were used, but only by birds from Beluga (see results); thus, they were less informative in characterizing differences between breeding sites in migratory timing than stopover sites used by birds from both breeding sites.

### Decision and option space

To evaluate the wind conditions shaping dowitcher migration strategies, we split their migration into three sections: (1) conditions at departure sites, (2) conditions-in-flight inside the Gulf of Alaska, where we expected the greatest potential differences between breeding sites due to high atmospheric heterogeneity, and (3) conditions-in-flight outside the Gulf of Alaska. Following Gill et al. 2014 [12], we used two temporally and spatially-separated states: ‘decision’ space and ‘option’ space. Decision space was defined by the period of time leading up to and including the departure date for the first migratory flight from an individual’s breeding site. We used this window of decision space to assess how cues derived from wind conditions might be integrated into migratory departure decision making. Option space was derived from in-flight GPS locations post departure and describes the route choice an individual made once aloft on its first migratory flight. For our purposes, it also included the total option space available: all other routes that an individual could have chosen, based on the routes that other birds from Beluga and King Salmon selected. To assess differences between the routes actually chosen and all potential route options, we included four route options for birds departing from King Salmon – flights to Cordova (AK), Northern California, Southern California, and Baja California – and five route options for birds departing from Beluga – flights to King Salmon (AK), Cordova (AK), Washington, Northern California, and Baja California.

### Wind data

To assess how wind conditions influenced both the decision and option spaces for dowitchers, we retrieved wind data from the European Center for Medium-Range Weather Forecasts ERA5 climate reanalysis dataset [36] *via* the Movebank Env-DATA system [37]. Hourly wind (e.g., the *u*- and *v*- wind components, which represent the east/west and north/south components of the wind vector, respectively) data are available at a 0.25° latitude-longitude scale from sea level to over 20,000 m above sea level. We extracted the daily values of four wind variables – maximum speed, mean speed, mean direction, and mean tailwind support – in order to match the frequency of location estimates from our transmitters. To evaluate conditions in the decision space, we created a decision window for each bird: the daily wind metrics at the closest grid cell to the last GPS-location estimate taken on the breeding grounds at 100, 750, and 1500 m above sea level (msl) for each of the five days leading up to departure. Wind at 850 millibars (1,170–1,590 msl) has been described as the likely altitude used by birds to determine their departure timing [8, 38], and wind at 100msl is close to sea level but above topographic features that would interfere with wind reanalysis data availability at sea level. We sampled at three altitudes to evaluate if dowitchers used different conditions to make their decisions. This analysis also allowed us to assess if the wind conditions on each day leading up to departure differed from the conditions on the departure day.

We calculated wind support (m/s) as:

$$\:ws\:=\:cos\:\left(atan2\right(u,\:v)\:-\:\theta\:\pi\:\:180\:)\:\times\:\:\sqrt{{u}^{2}}\:\:\times\:\:\sqrt{{v}^{2}}\:\:$$

where *u* and *v* represent the east/west and north/south components of the wind vector, respectively, and *θ* represents the direction of an individual’s travel to their first flight destination in radians.

To evaluate wind support in the option space, we simulated tracks between each breeding site and each potential first-flight destination in the R package “sf” [39], and sampled points along the tracks every 60 km, the highest spatial resolution for wind conditions available with minimal uncertainty within the ERA5 dataset. We calculated tailwind conditions at each sampled point along each of the nine potential routes for all days that birds were in flight to compare tailwind access between routes and departure sites, as well as conditions for all days throughout the total migratory window, 30 Jun – 1 Aug. Some of the transmitters provided altitudinal data in flight (*n* = 12), and thus provided a range of flight altitudes. However, shorebirds dynamically alter their flight altitudes over the course of a migratory flight, and we therefore calculated wind support in the Gulf of Alaska at three different altitudes above sea level at which shorebirds are known to fly: 500, 1000, and 1500msl [40].

### Statistical analysis

All analyses were done in the R Programming Environment [41] in the package “lme4” [42].

1. *Migratory timing*: We ran four non-interactive GLMs with site as the sole predictor variable. The response variable for each model was the date of (i) departure from the breeding site or arrival to (ii) latitude 40.8°N (i.e., northern California), (iii) latitude 32.7°N (i.e., southern California), or (iv) the final nonbreeding site.
2. *Wind in decision space*: We tested for collinearity among predictor variables and chose variables that did not exhibit significant collinearity. We ran three additive global models for each potential altitude (100, 750, and 1500msl) that included five predictor variables: maximum daily wind speed, mean daily tailwind speed, mean daily wind direction, site, and a single two-way interaction term between pairs of these variables. The response variable was the binary (whether or not a bird departed) and the random effect was individual, as each bird had six separate potential/real departure dates. We compared models using Akaike’s Information Criterion values corrected for small sample sizes (AIC_c_) [43]. To address the two-day uncertainty in eight of the departure dates (Table 1), we ran these three model sets for both sets of possible departure days. We assessed model support based on model weights and whether predictor variables had parameter estimates (± 95% confidence intervals; [44]) that did not overlap zero (Table 2). Additionally, we ran two GLMMs to determine the overall differences in wind conditions between sites, regardless of departure day, with site as the response variable and two predictor variables: maximum daily wind speed and mean daily wind direction at each of the three altitudes. This helped characterize how wind conditions at each site differed across the departure windows.
3. *Wind in option space*: We similarly ran three global models to assess wind conditions in the option space – one for each potential flight altitude (500, 1000, 1500msl) – which included four predictor variables: potential route (9 options), departure day of tracked bird (true/false), route and departure “match” (true/false: if the route was flown on a given day), and location (inside or outside the Gulf of Alaska). The response variable was mean daily tailwind speed for all sampled points along each route. Like our analysis of decision space, we assessed model support based on model weights and whether predictor variables had parameter estimates (± 95% confidence intervals; [44]) that did not overlap zero (Table 3). Additionally, we used a Tukey post-hoc test to compare wind conditions between each unique pair of routes.

**Table 1:**
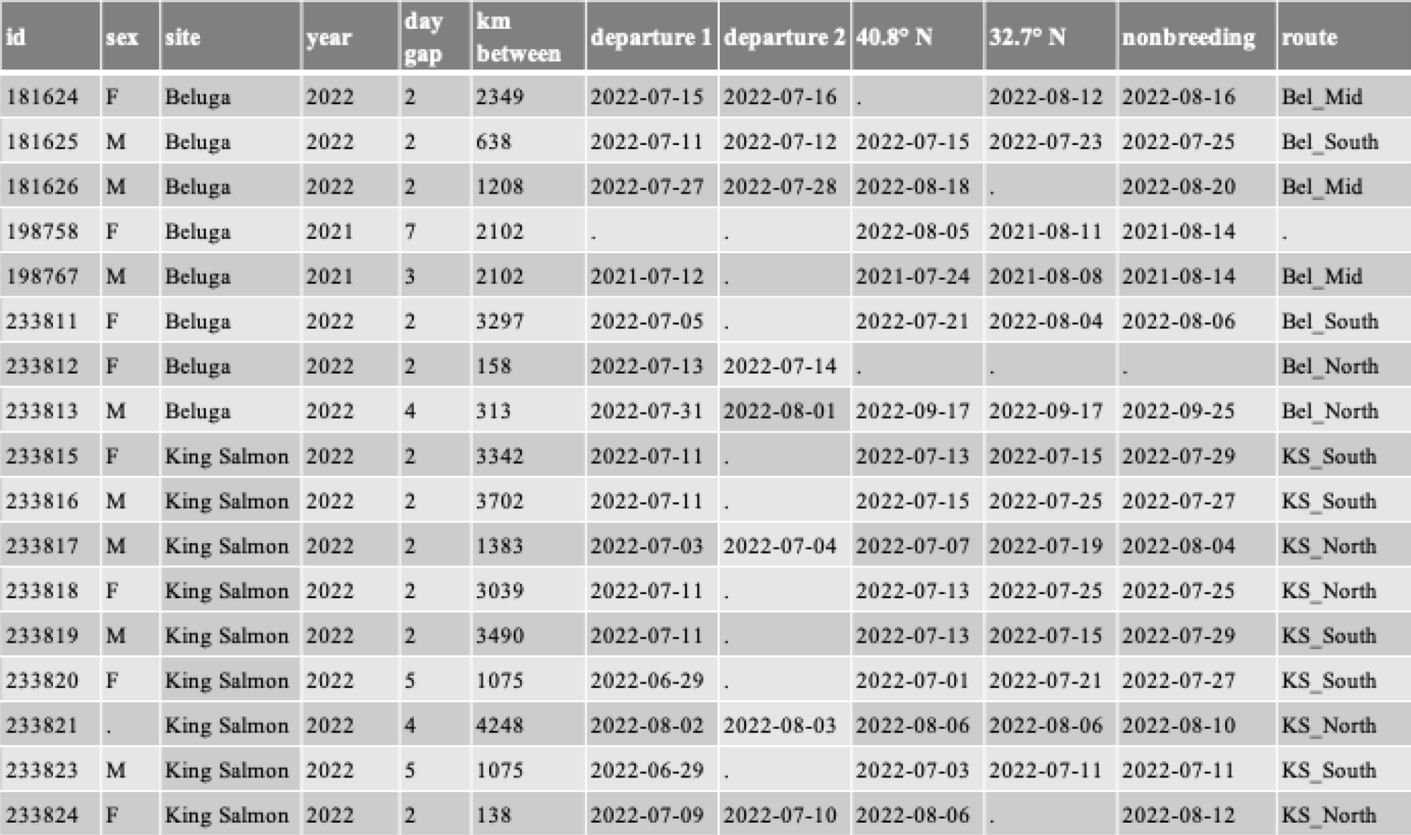
Tracked individuals used in analysis. ‘id’ refers to individual bird, ‘day gap’ refers to the number of days between the last fix on the breeding grounds and the first fix away from that region, ‘km between’ is the number of kilometers between the breeding grounds and the first fix away from that region, ‘departure 1 & 2’ are the possible dates of departure, ‘40.8’ and ‘32.7’ are the dates the birds were tracked south of those respective latitudes, ‘nonbreeding’ is arrival date to nonbreeding site, and ‘route’ is the route in the Gulf of Alaska the individual selected

**Table 2:** Model selection in decision space. Top three models for each elevation, ranked by AICc. Predictor variables are normalized daily maximum wind speed, daily wind direction, daily tailwind, and site. Response variable is the log-odds of a bird departing

(Intercept)	Daily max speed	Daily direction	Daily tailwind	Site	df	logLik	AICc	Delta AIC_c_	msl
-4.71	NA	NA	5.61	NA	3	-12.54	31.49	0	100
-6.82	-1.97	NA	5.51	NA	4	-11.60	31.89	0.40	100
-8.27	-2.94	NA	5.46	+	5	-11.26	33.58	2.09	100
-2.01	-1.51	-0.98	NA	NA	4	-30.17	68.78	0	1500
-2.64	-1.08	NA	1.47	NA	4	-31.00	70.42	0	750
-2.22	-1.53	-0.97	NA	+	5	-30.00	70.59	1.81	1500
-2.01	-1.39	-0.81	0.267	NA	5	-30.01	71.00	1.92	1500
-2.56	-1.02	-0.23	1.30	NA	5	-30.63	71.92	1.50	750
-2.71	-1.12	NA	1.44	+	5	-30.97	73.00	2.18	750

**Table 3:** Model selection in option space. Top three models for each elevation, ranked by AICc. Predictor variables are location in/out of the Gulf of Alaska, match (if the route was flown on a departure day), and route. Response variable is normalized daily mean tailwind

(Intercept)	GOA	Match	Route	df	logLik	AICc	Delta AIC_c_	msl
-1.60	+	+	+	12	-26869.90	53763.84	0	1000
-1.54	+	NA	+	11	-26878.15	53778.33	14.49	1000
-3.14	NA	+	+	11	-27026.31	54074.65	310.81	1000
-2.33	+	+	+	12	-28315.45	56654.93	0	1500
-2.29	+	NA	+	11	-28318.69	56659.41	4.48	1500
-3.51	NA	+	+	11	-28402.40	56826.84	171.90	1500
-0.05	+	+	+	12	-31124.12	62272.26	0	500
-0.02	+	NA	+	11	-31126.88	62275.79	3.53	500
-1.98	NA	+	+	11	-31396.64	62815.31	543.04	500

## Results

### Transmitter deployment and migratory routes

We deployed 12 transmitters in 2021 in Beluga, but many of these transmitters failed completely (*n* = 4) or had such large gaps in fixes that they were unusable for our analyses (*n* = 6). This left two usable tracks, one of which did not capture an accurate departure day from Alaska but was used in our other analyses. We deployed 20 additional transmitters in 2022: 10 on birds breeding in Beluga and 10 on birds breeding in King Salmon. Out of the 20 transmitters deployed in 2022, 9 from King Salmon and 6 from Beluga provided full fall migratory tracks. One bird from Beluga was tracked in both 2021 and 2022, and we were able to use both years of data. Of the transmitters that did not provide movement data, two transmitters from Beluga remained stationary throughout the fall, which indicated that they fell off the birds or that the birds died, while three transmitters failed to transmit data. Our total sample size was therefore 17 southbound migratory tracks: 9 from King Salmon and 8 from Beluga (Table 1).

**Fig. 2:**
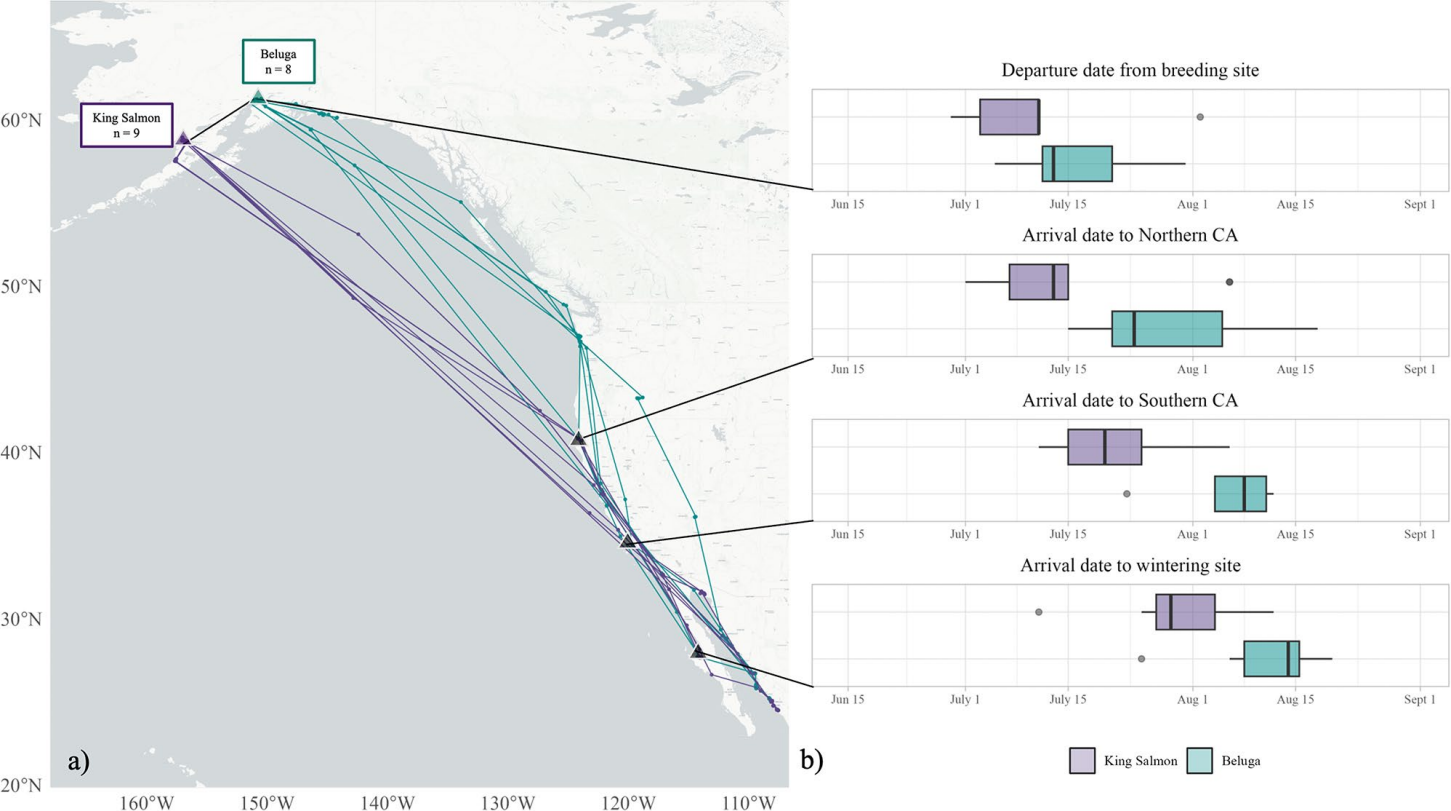
(**a**) Short-billed Dowitchers tracked from two Alaskan breeding sites during Fall migration. (**b**) Boxplots depict timing of departure from breeding grounds and arrival to latitudes associated with stopover sites and wintering sites: Arcata Marsh in northern California, San Diego Bay in southern California, and wintering sites along coastal Mexico.

All dowitchers used the Pacific Americas Flyway during their southbound migrations, but birds from King Salmon took a more westerly and oversea route than birds from Beluga (Fig. 2a). Upon departing from coastal Alaska, dowitchers departing from King Salmon flew 2388 ± 1469 km (values represent mean ± SD unless otherwise noted) on their first flight, while dowitchers from Beluga flew 1438 ± 1178 km. Dowitchers from King Salmon did not use any stopover sites until they reached the coast of California, at which point they stopped at one of just three sites: Arcata Marsh (*n* = 3), San Francisco Bay (*n* = 3), or San Diego Bay (*n* = 3). Following their first flight, dowitchers departing Beluga stopped at four different sites along the coast of the Gulf of Alaska and northwestern United States: Middleton Island, Alaska (*n* = 1), Cordova, Alaska (*n* = 2); Gray’s Harbor, Washington (*n* = 4), and Arcata Marsh (*n* = 1). Stopover frequency and duration differed between the breeding sites, with dowitchers from Beluga using an average of 2.1 ± 0.9 stopover sites and spending an average of 17.9 ± 10.1 total days stopped over, and dowitchers from King Salmon using an average of 1.2 ± 0.4 stopover sites and spending an average of 12.7 ± 7.8 total days stopped over. Two birds (from different mated pairs) were tracked flying together from King Salmon directly to San Francisco Bay, covering 3400 km in three days. Two birds from Beluga used the interior-Pacific Americas Flyway after making their first landfall, stopping in California’s Central Valley and the Great Basin, respectively; all birds from King Salmon remained along the coast after their first landfall. The dowitcher tracked from Beluga for two consecutive years used the same route from Alaska to coastal Washington in both years. All dowitchers wintered in Mexico, either on the Pacific Coast of Baja California or on the coastal mainland from the upper Gulf of California south to Sinaloa.

### Migratory timing

All birds departed their breeding grounds between 30 June – 1 August (hereafter, “migratory window”), with 8 leaving between 10–14 July. We therefore found no significant difference in departure timing between the two sites (ß= -6.6, SE = 4.9, *p* = 0.2). However, dowitchers departing from King Salmon crossed both 40.8° in northern California and 32.7° in southern California 22 days earlier than birds from Beluga (ß= -22.3, SE = 8.5, *p* = 0.04 and ß= -22.5, SE = 7.4, *p* = 0.01, respectively). As a result, birds from King Salmon preceded birds from Beluga to their final nonbreeding sites by an average of 18.5 ± 7.3 days (ß= -18.5, SE = 7.3, *p* = 0.02; Fig. 2b).

### Flight altitude

Transmitters that captured altitudinal data (*n* = 12 transmitters) recorded birds flying between 111 and 2386 msl, (µ = 993 ± 634 msl, *n* = 21 fixes). Only four of the points with altitudinal data were recorded during flights above the Gulf of Alaska, where the altitudes ranged from 111 to 1977 msl (µ = 1077 msl).

### Wind conditions in decision space

**Fig. 3:**
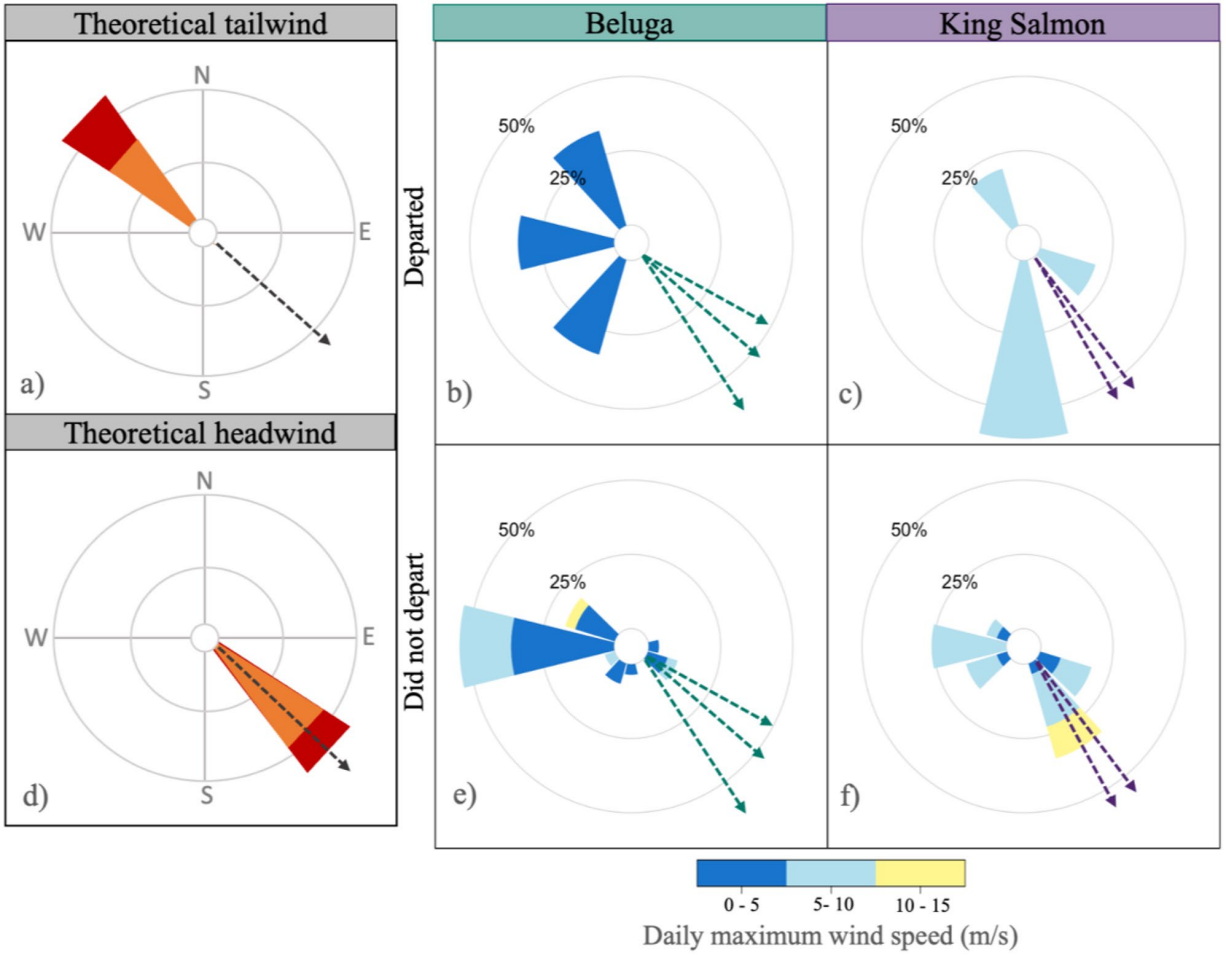
Wind roses depicting wind conditions on the breeding grounds at 100 msl. Warm colors represent faster speed (m/s) and the length of spoke represents the frequency of wind direction, with dashed lines depicting bird direction of travel. (**a**) Theoretical northwesterly wind blowing towards the theoretical direction of travel southeast. (**b**) & (**c**) Wind conditions on days when birds departed. (**d**) Theoretical southwesterly wind blowing against the theoretical direction of travel. (**d** & **e**) Conditions on the five days preceding departure

The most well-supported model for predicting departures in the decision space for all possible departure days included mean daily tailwind at 100msl (AIC_c_ = 31.48, Table 2). No model with conditions at any other altitude was within 4 AIC_c_ units of the most well-supported model. Although maximum wind speed at 100msl was higher at King Salmon than Beluga, there were no significant differences in mean daily tailwind between sites, and winds were unprofitable throughout the decision window no matter from which site an individual departed (Beluga: -0.70 ± 0.70 m/s; King Salmon: -1.08, ± 2.67 m/s). There were 8 birds with multiple departure day options, because of gaps between GPS fixes. Depending on the potential departure day used, birds departed on days with either more (scenario one: ß = 1.44, SE = 0.78, *p* = 0.06) or less (scenario two: ß = -1.28, SE = 0.56, *p* = 0.02; Fig. 3) profitable conditions than during the preceding 5-day window.

### Wind support in option space

**Fig. 4:**
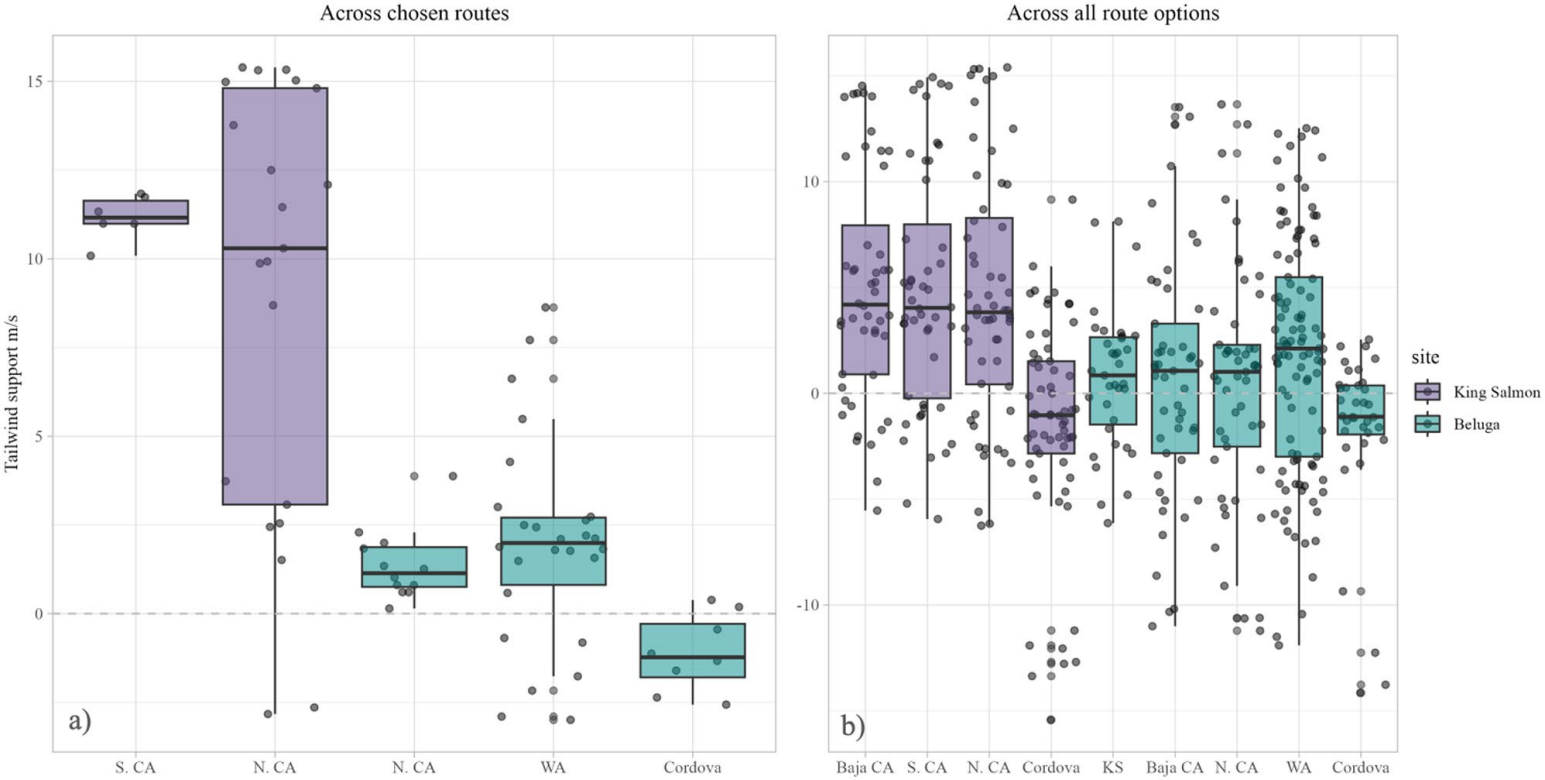
Tailwind support on the days birds were aloft in the Gulf of Alaska, at 1000 msl. (**a**) Tailwind support on selected routes on the days they were flown (chosen option space). (**b**) Tailwind support across all routes selected on all departure days (total option space)

The most well-supported model explaining how tailwinds differed in the option space included the variable denoting whether the route was both selected (by any bird) and flown on the day birds were aloft, as well as the variable denoting whether the point was within the Gulf of Alaska or not. The models performed best at 1000msl (AIC_c_ = 53763.83, Table 3), which aligned with the altitudinal data reported from transmitters. The Tukey post-hoc test showed that the dowitchers departing from King Salmon benefitted from greater tailwind support than birds departing from Beluga across all chosen routes on selected departure days within the Gulf of Alaska (Fig. 4a). Those departing from King Salmon on routes bound for Northern and Southern California experienced 10 and 12 m/s more tailwind than routes from Beluga flying to Cordova, respectively; 7 and 9 m/s more than those flying from Beluga to Washington; and 8 and 10 m/s more than those flying from Beluga to Northern California. Conditions did not differ significantly between chosen routes from King Salmon to Northern and Southern California, nor between chosen routes from Beluga to Cordova, Washington, or Northern California.

**Fig. 5:**
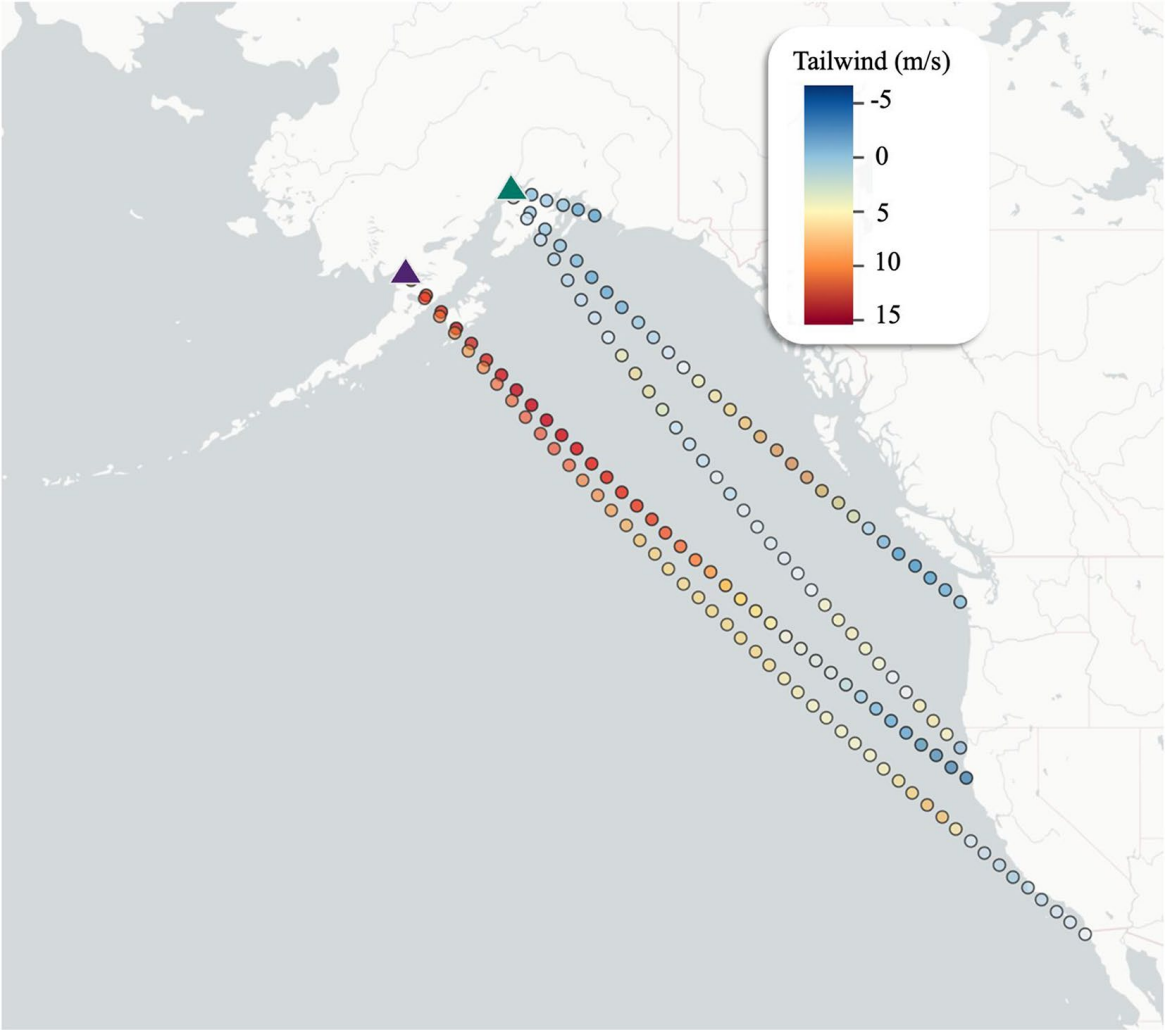
Average daily tailwind support at sampled points along selected routes on days dowitchers were in flight. Higher values shown in red indicate stronger tailwinds, and lower values in blue indicate weaker tailwinds or lack of tailwind.

Wind conditions between chosen routes across all departure days (not just the matching route and day), showed similar trends but less supportive tailwinds overall (Fig. 4b). Those departing from King Salmon on the two selected routes (to Northern and Southern California) experienced 6 m/s more tailwind than those from Beluga flying to Cordova, 3 m/s more than those flying from Beluga to Washington, and 4 m/s more those flying from Beluga to Northern California (Fig. 5). On the four simulated routes that were not selected by any tracked individual, tailwind support varied within the Gulf of Alaska on departure days. A bird electing to fly from Beluga to King Salmon would have benefitted from 2 m/s more tailwind than during a flight from Beluga to Cordova, but the same support compared to other selected routes from Beluga. Alternatively, a bird electing to fly from King Salmon to Cordova would have experienced 6 m/s less tailwind support than if it had flown to either northern or southern California from King Salmon. Direct routes from both King Salmon and Beluga to Baja California would have offered similarly supportive tailwind to birds departing from their respective locations on other selected routes, though those departing from King Salmon would have experienced 4 m/s more support than those departing from Beluga.

Finally, tailwind conditions outside the Gulf of Alaska at 1000msl were 1 m/s more profitable than those inside the Gulf of Alaska across selected routes and all departure days (ß = 1.00, SE = 0.32, *p* < 0.001). However, this difference decreased on days birds were actually aloft: along the selected routes on the days birds departed, tailwinds outside the Gulf of Alaska were just 0.11 m/s greater than those inside the Gulf of Alaska (ß = 0.89, SE = 0.31, *p* = 0.004). Further, there was a difference in tailwind support outside of the Gulf of Alaska between sites: birds departing from King Salmon experienced an average of 2.5 m/s more support than those from Beluga along selected routes and departure days (ß = 2.53, SE = 0.27, *p* < 0.001).

## Discussion

Ecological barriers are often characterized as a cause of migratory divides, as migrants adjust their routes to avoid the challenges these barriers present. This concept is challenged when temporally variable environmental conditions can help migrants cross a barrier, potentially making it a corridor [8]. In this study, we compared the southbound migration strategies of an understudied species of shorebird from two different breeding sites that span the edge of a dynamic ecological barrier – the Gulf of Alaska. Between our two study sites, migratory timing and routes differed, while total distance traveled and nonbreeding destinations did not. Dowitchers did not show a difference in their departure timing from our two study sites, but those from the Alaska Peninsula arrived to the nonbreeding grounds an average of 19 days earlier than their counterparts from Cook Inlet. This difference in arrival timing inspired our investigation into the role wind plays in shaping a spatiotemporal migratory divide between the sites while crossing the Gulf of Alaska. Tailwind access at both breeding sites at departure was low, and dowitchers departed for fall migration on days with little, if any, wind support. Once en route, access to tailwinds differed substantially between routes within the Gulf of Alaska. Birds that departed from the Alaska Peninsula took longer, more exposed flights across the Gulf of Alaska and experienced greater tailwind support along those routes than birds that departed from Cook Inlet. This difference in wind support, along with the number and duration of stopovers used, is a potential explanation for the marked difference in migratory duration between birds at the two breeding sites, suggesting that crossing formidable barriers directly can, under some conditions, offer a corridor to the nonbreeding grounds. Our study thus offers a fine-scale investigation of migratory birds departing Alaska, and counters past efforts utilizing weather surveillance radars, which have suggested that birds departing both the Alaska Peninsula and Cook Inlet generally fly northeast at the initiation of fall migration [45].

### Integrating wind cues to inform decision and option space

Many studies have provided evidence about the importance of tailwinds as a departure cue for aerial migrations [46, 47], especially at the edges of ecological barriers [11, 13]. Tailwinds may be most strongly selected for at departure when they are predictive of the wind regimes found farther along the migration route. Dowitchers had relatively little access to tailwinds at departure at either study site and may even have departed on days with slightly less wind support than preceding days. While access to tailwinds did not differ between sites, once aloft in the Gulf of Alaska, dowitchers from our two study sites experienced a range of tailwind support along their selected routes, with almost all birds gaining access to tailwinds en route. These results suggest that potentially: (1) even slight tailwinds at the two breeding sites are predictive of beneficial winds over the Gulf of Alaska, (2) dowitchers may be able to integrate other weather-related cues about when to initiate migration that are informative of atmospheric conditions, such as barometric pressure [10], or finally, (3) information derived from other sources known to shape migratory choices, such as photoperiod [48], temperature [49], food availability [50], and social cues [51], outweigh the information gleaned from wind conditions on the breeding grounds. If the third alternative is true, then the decision to initiate migration would likely be decoupled from route choice, with the latter determined once in flight.

Almost all tracked dowitchers selected routes in the option space with at least some tailwind support and experienced more support along their chosen route than they would have along a different route or on a different day. This could imply that dowitchers have the ability to discern between wind conditions and flexibly alter their route direction, or potentially have knowledge of specific, beneficial routes used in migrations past. The exceptions were two tracked birds that flew the short distance from Beluga to Cordova into headwinds. These individuals would have benefitted from tailwind support had they chosen any of the other routes taken by dowitchers departing Beluga, or even had they flown to King Salmon. Their decision to fly to Cordova indicates that at least on some flights, birds departing from Beluga likely integrate other cues besides wind to decide the distance and destination of their first flight: possibilities include body condition, social cues, or, because of the relatively accessible coastline, knowledge of specific stopover sites [58].

More generally, tailwinds were consistently more profitable on the two selected migratory routes from King Salmon than the three routes from Beluga. This was true across all days when birds were aloft in the Gulf of Alaska, during which tailwind support along routes from King Salmon was an average of 3 m/s greater than Beluga routes. It was also true along the specific routes individuals chose, where birds departing from King Salmon experienced an average of 5.5 m/s more tailwind support than those flying from Beluga on their selected routes. Increase in tailwind support for birds results in greater groundspeeds [52], which can in turn lower the energetic cost of flight by both shortening migratory duration and reducing energy expended while aloft [53, 54]. This greater access to tailwinds, coupled with a dearth of stopover opportunities en route, could explain the earlier arrival of King Salmon dowitchers to nonbreeding sites. It could also suggest that birds departing from King Salmon expend less energy than those from Beluga during the first leg of migration.

### The downstream effect of differential support: stopover sites and arrival timing

As post-breeding migration is typically thought to be under less time selection than pre-breeding migration [55], the tradeoffs involved in nonbreeding site arrival timing may not be as apparent as to a breeding site. The benefits of arriving early — for instance, minimizing time during migration to maximize time at the nonbreeding site — therefore might be decoupled from the benefits of arriving before other individuals. Simply arriving early may shape an individual’s ability to access food, depending on the phenology of resources at the destination [56]. Arriving before other individuals could also influence intra- and inter- specific interactions, including competition for territories at wintering sites [57, 58] or the potential benefits of congregating in large numbers for protection from predation [59].

Depending on a species’ degree of sociality and the temporal variation in resource availability, the dynamics of intra- and interspecific interactions may present a tradeoff. Relatively little is known about dowitchers at their nonbreeding sites, but an observed increase in aggressive behavior between individuals on the Massachusetts coast during southbound migration was linked with high patchiness in the distribution of a preferred food, horseshoe crab (*Limulus polyphemus*) eggs [60]. The nonbreeding sites used by dowitchers along Mexico’s mainland coast are also used by hundreds of thousands of individuals from 28 other species of shorebirds [61]. This presents the opportunity for both competition and mutualism in mixed flocks, the relative balance of which may vary as the number of individuals present fluctuate throughout the fall and winter months. The timing of food resource availability in sites used by shorebirds along the Pacific Flyway has also not been tracked during late summer to fall, but the availability of Polychaete worms (a favored dowitcher food item) varies temporally in this region [62, 63]. Such temporal variation in resource availability suggests that timing of arrival at nonbreeding sites might benefit individuals needing to rapidly refuel after a demanding breeding season and migratory journey.

### A tenuous corridor across a barrier

Our study provides additional support for the idea that a migrant crossing the Pacific Ocean may gain access to an “ecological corridor” depending on where its route falls along the gradient of wind support that characterizes the region from west to east. Crossing a vast oceanic barrier may have extreme consequences, as the number of ‘bailout’ options for resting or refueling are greatly reduced for migrants unable to stop on water (i.e., shorebirds and songbirds) [29]. Likewise, a migratory strategy with more stopovers can increase an individual’s opportunity to glean information, rest, refuel, and wait out inclement weather while en route [64]. For a migrant employing this frequent stopover strategy, body condition upon arrival to a nonbreeding site might hinge more upon resource availability at selected stopover sites than the relative conditions encountered while in flight. However, a trans-oceanic strategy can have substantial benefits. Fewer stopovers likely confer lower exposure to predators and pathogens [8, 65], as well as less time overall spent migrating [66], a period often constituting one of the riskiest parts of a migrant’s annual cycle [67, 68]. Summer storms in the Gulf of Alaska are consistently less intense and frequent than in other seasons, and fall storms show low variability between years [69]. If favorable conditions across a barrier are reliable across the migratory window within and between years, a canalized strategy to take advantage of them might be far less risky than complete avoidance of the barrier altogether.

The Gulf of Alaska supports other migratory bird crossings in addition to those of dowitchers: Marbled Godwits (*Limosa fedoa*) tracked from the Alaska Peninsula [70] and Whimbrel (*Numenius phaeopus*) from further north and east in Alaska [71] followed similar routes to those of King Salmon dowitchers on their way to nonbreeding sites along the Pacific Coast. Middleton Island – an isolated refuge in the northern Gulf of Alaska – hosts at least 173 migratory bird species stopping over during southbound migration, most of which are passerines that likely time their migratory efforts across the Gulf of Alaska with northwesterly (southeast blowing) winds [72]. Other tracked species confronting the Gulf of Alaska employ avoidance strategies, such as both Glaucus-winged Gulls (*Larus glaucescens*) [73] and Sooty Fox Sparrows (*Passerella iliaca unalaschcensis*) [74], which use coastal sites during migration between Alaska and central-California. Such diversity in migration strategies indeed suggests that the Pacific Basin is a ‘theater’ for bird migration and that access to, and the ability to make use of, supportive winds is key for understanding how different populations and species cross the region.

Although our one individual tracked across two years exhibited similar migratory decisions across years, with just one year of tracking data for most individuals and a small sample size overall, we are unable to generalize about the importance of wind as a migratory cue for dowitchers, nor about the persistence of a migratory divide between breeding sites. Individuals of other shorebird species are capable of changing their migration strategies between years [70, 71], and migratory raptors can improve their ability to compensate for wind drift with age [75], meaning there is likely variation among individuals and years in the ability of dowitchers to integrate cues and capitalize on tailwinds. However, of all tracking studies conducted across the Gulf of Alaska, ours is the only one of which we are aware that demonstrates marked intraspecific differences in migration strategy across a species’ breeding range. At least for dowitchers, this implies that there may exist flexibility in route choice across the Gulf of Alaska and that, in other years and with different conditions, individuals might exhibit different migratory decisions [8].

## Conclusions

A looming tradeoff for a migration strategy that relies upon temporally fluctuating conditions is future uncertainty in the face of climate change. Long-term datasets for global wind patterns are not nearly as robust as they are for temperature and precipitation, but available data suggest that wind conditions are changing rapidly and overall trending toward a “global stilling” [76, 77]. The change is not homogenous: the Aleutian Low pressure system, which is the main driver of wind in the Gulf of Alaska and weather across much of the western United States, is expected to intensify in its extremes [78], possibly strengthening large-scale winds in the Pacific Basin. Hurricanes and tropical storms are forming earlier and with more strength [79], both impacting migratory flyways and acting in concert with sea level rise [80] and human development [81] to alter coastlines. As a result, long-distance and long-lived migrants that depend upon once-consistent conditions are now being exposed to rapid change over the course of their lifetimes [82]. A migration strategy that relies upon a nonstop flight across a dynamic region like the Gulf of Alaska might become riskier, with success hinging upon a narrow set of beneficial conditions, than strategies that include more stopover options. To respond effectively to changing conditions, a migrant’s ability to integrate multiple cues into migratory decisions will become increasingly important. Ultimately, individual- and population-level variation in migration strategy may therefore provide a glimpse into the adaptability of long-distance migratory species.

## Data Availability

The datasets supporting the conclusions of this article are available in the figshare repository, https://figshare.com/s/8fc9648cd4ffceda70bd.
